# Macro-reentrant atrial tachycardia after tricuspid or mitral valve surgery: is there difference in electrophysiological characteristics and effectiveness of catheter ablation?

**DOI:** 10.1186/s12872-021-02368-w

**Published:** 2021-11-12

**Authors:** Xin-hua Wang, Ling-cong Kong, Tian Shuang, Zheng Li, Jun Pu

**Affiliations:** Department of Cardiology, Ren Ji Hospital, Shanghai Jiao Tong University School of Medicine, 160 Pujian Road, Shanghai, 200127 China

**Keywords:** Macro-reentrant atrial tachycardia, Atrial flutter, Tricuspid valve surgery, Mitral valve surgery, Catheter ablation

## Abstract

**Background:**

Macro-reentrant atrial tachycardias (MATs) are a common complication after cardiac valve surgery. The MAT types and the effectiveness of MAT ablation might differ after different valve surgery. Data comparing the electrophysiological characteristics and the ablation results of MAT post-tricuspid or mitral valve surgery are limited.

**Methods:**

Forty-eight patients (29 males, age 56.1 ± 13.3 years) with MAT after valve surgery were assigned to tricuspid valve (TV) group (n = 18) and mitral valve (MV) group (n = 30). MATs were mapped and ablated guided by a three-dimensional navigation system. The one-year clinical effectiveness was compared in two groups.

**Results:**

Nineteen MATs were documented in TV group, including 16 cavo-tricuspid isthmus (CTI)-dependent AFL and 3 other MATs at right atrial (RA) free wall, RA septum and left atrial (LA) roof. Thirty-nine MATs were identified in MV group, including15 CTI-dependent AFL, 8 RA free wall scar-related, 2 RA septum scar-related, 8 peri-mitral flutter, 3 LA roof-dependent, 2 LA anterior scar-related, and 1 right pulmonary vein-related MAT. Compared with TV group, MV group had significantly lower prevalence of CTI-dependent AFL (38.5% vs. 84.2%), higher prevalence of left atrial MAT (35.9 vs.5.3%) and higher proportion of patients with left atrial MAT (40 vs. 5.6%), *P* = 0.02, 0.01 and 0.01, respectively. The acute success rate of MAT ablation (100 vs. 93.3%) and the one-year freedom from atrial tachy-arrhythmias (72.2 vs. 76.5%) was comparable in TV and MV group. No predictor for recurrence was identified.

**Conclusion:**

Although the types of MATs differed significantly in patients with prior TV or MV surgery, the acute and mid-term effectiveness of MAT ablation was comparable in two groups.

*Trial registration:* This study was registered as a part of EARLY-MYO-AF clinical trial at the website ClinicalTrials. gov (NCT04512222).

**Supplementary Information:**

The online version contains supplementary material available at 10.1186/s12872-021-02368-w.

## Introduction

Macro-reentrant atrial tachycardias (MATs) can occur in a considerable proportion of patients early or late after cardiac valve surgery [1]. Atriotomy, suture and fibrotic scars provide an ideal substrate for MATs [2, 3]. Due to the complex substrate and coexistence of multiple reentries, catheter ablation of MATs in this scenario is often challenging and is associated with compromised long-term results [4–6]. In contrast to tricuspid valve surgery, mitral valve surgery involves inter-atrial septum incision and left atriotomy rather than right atriotomy, which might produce septal and left atrial MATs instead of right atrial MATs [7–9]. However, there is paucity of data regarding the difference in the electrophysiological characteristics of MATs after tricuspid or mitral valve surgery, and the effectiveness of MAT ablation in these patients has not been adequately investigated.

This retrospective study was carried out to explore the electrophysiological characteristics of MAT and the effectiveness of MAT ablation in patients with tricuspid or mitral valve surgery. Of note, the surgical technique might vary between centers and therefore, the findings of this study might not be generalized to all patients with status post valve surgery arrhythmias.

## Method

### Patient enrollment

Forty-eight patients (29 males, average age 56.1 ± 13.3 years) with the history of tricuspid or mitral valve surgery in Ren Ji Hospital, Shanghai Jiao Tong University School of Medicine were enrolled consecutively to undergo catheter ablation of MAT from April 2013 to December 2019. The diagnosis of MAT was primarily based on rapid and regular atrial activity with monomorphic P wave (or flutter wave) on standard twelve-lead electrocardiograms (ECG) and consistent atrial activation sequence on endocardial recording. The inclusion criteria were: repetitive or sustaining MAT occurred > 2 months post-valve surgery; failure of one or more antiarrhythmic drugs (AADs) therapy or unwilling to take AADs. The exclusion criteria were: prior ablation of atrial tachycardia (AT) or atrial fibrillation (AF); malfunction of valve prosthesis; presence of left atrial thrombus detected by transesophageal echocardiography; decompensated heart failure; concomitant AF at the time of enrollment; unable to provide written informed consent. All enrolled patients were divided into two groups according to the type of valve surgery: tricuspid valve (TV) group and mitral valve (MV) group.

### Electrophysiological study

The procedure was performed in a fasting state under conscious sedation with continuous infusion of fentanyl and midazolam. All AADs were discontinued for at least five half-lives except amiodarone (the latter was suspended for > 1 month prior to ablation). Oral anticoagulation with therapeutic warfarin (target international normalized ratio [INR] 2.0–3.0) was continued in each patient. Intravenous unfractionated heparin dose was titrated to maintain an activated clotting time (ACT) of 300–350 s throughout the procedure. A decapolar deflectable mapping catheter was positioned in the coronary sinus (CS) via left femoral vein access, ensuring the proximal electrode pair positioned at the CS ostium. Two 8.5 French SL-1 sheathes (St. Jude Medical, MN) were advanced into the right atrium (RA) via right femoral vein access, or were introduced into the left atrium (LA) via transseptal catheterization when the left atrial MAT was considered. A duo-decapolar mapping catheter (PentaRay, Biosense Webster, CA) and a 3.5 mm saline-irrigated ablation catheter (Thermo-cool STSF, Biosense Webster, CA) was inserted through the long sheathes for mapping and ablation under the guidance of CARTO (Biosense Webster, CA). Endocardial bipolar electrograms were filtered at band-pass 30–500 Hz and were measured at a sweep speed of 100 mm/s (Prucka CardioLab EP System, GE Healthcare). If the MAT was not spontaneously initiated, it was induced by CS burst pacing with isoproterenol infusion (2-5ug/min).

### Differential diagnosis of MAT

The diagnosis of MAT was based on the continuous activation pattern with the “early meets late” phenomenon and the whole activation time of the target atrium covering over 90% of the MAT cycle length (CL) [7, 10, 11]. Additionally, a post-pacing interval (PPI) not exceeding MAT-CL by more than 30 ms (ms) after entrainment at two separate sites favored the establishment of macro-reentrant mechanism [7, 10, 11] (Additional file 1: Fig. S1).

**Fig. 1:**
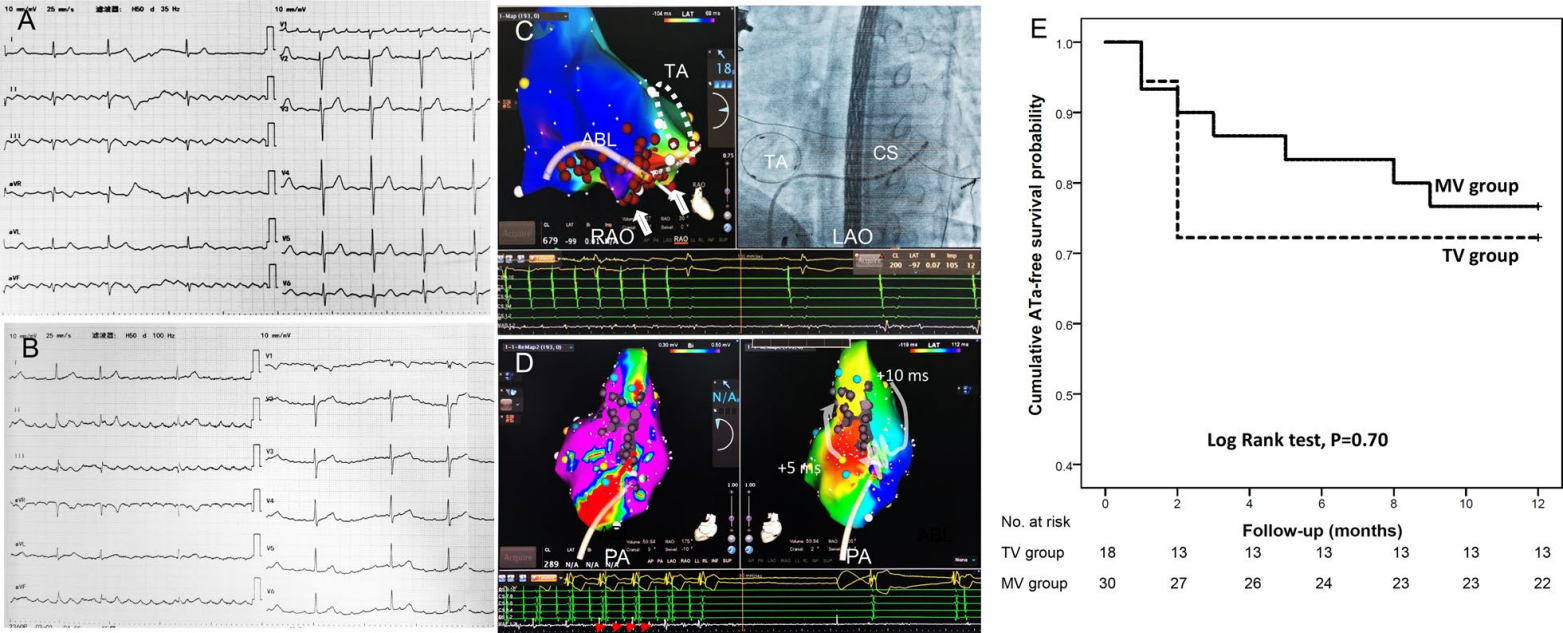
Right atrial MAT ablation. In **A** the flutter wave of counterclockwise CTI-dependent AFL was negative in inferior limb leads (II, III and aVF), positive in precordial lead V1, and progressively became shallow negative from precordial lead V2 to V6. In **B** the flutter wave of clockwise CTI-dependent AFL was positive in inferior limb leads, negative in precordial lead V1, and progressively became positive in precordial lead V2 to V6. In **C** counterclockwise AFL was terminated by CTI linear ablation after tricuspid valve bio-prosthesis implantation. Of note, two pouches were found in proximity to the tricuspid annulus (TA) and at the mid-portion of the CTI area (white arrows). Extensive and prolonged RF energy delivery was needed to interrupt AFL and achieve CTI block. The dotted circle represented the tricuspid annulus (TA). In **D** right atriotomy-related macro-reentry was identified to around the scar line (gray dots line), entrainment pacing at either side of the scar line produced a matched PPI. The critical isthmus was found between the inferior border of the scar and the inferior vena cava, where short linear RF ablation terminated the tachycardia and rendered it non-inducible. Of note, the mid-diastolic, low-voltage and fractionated bipolar potentials were recorded at the critical isthmus (red arrows). In **E** the ATa-free survival probability was compared in MV group (solid line) and TV group (dotted line), *P* = 0.70 by Log-Rank test. *RAO* right anterior oblique view, *LAO* left anterior obliqueview, *PA* posterior-anterior view, *ATa* atrial tachyarrhythmia, *PPI* post-pacing interval, *LA* the left atrium, *CS* coronary sinus

MAT of right or left atrial origin was differentiated according to: i) the morphology of P or flutter wave on the surface ECG; ii) the CS activation sequence; iii) pacing entrainment from cavo-tricuspid isthmus (CTI), proximal and distal CS. The characteristic morphology of flutter wave in inferior limb leads (II, III, aVF) and precordial leads V1-V6 (Fig. 1A, B), the “proximal to distal” CS activation sequence and a PPI ≤ 30 ms longer than the MAT-CL after pacing entrainment at CTI rather than the proximal or distal CS favored the diagnosis of CTI-dependent AFL. Whereas the positive flutter wave in inferior leads as well as precordial leads, the “distal to proximal” CS activation sequence and a better PPI after pacing entrainment at the distal CS rather than the proximal CS or CTI were consistent with left atrial MAT.

**Fig. 2:**
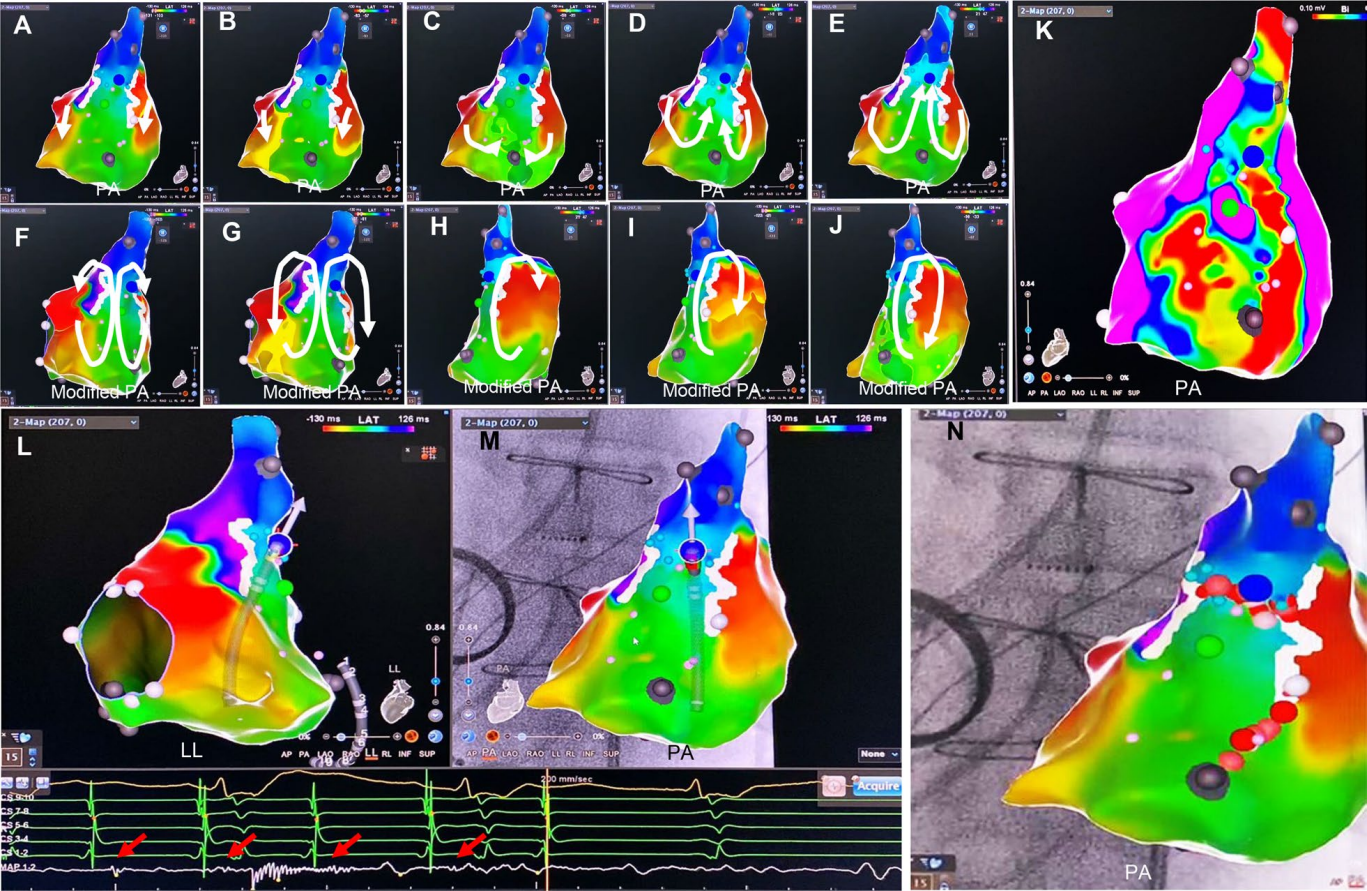
“Figure-of-eight” right atrial MAT ablation after mitral valve prosthesis replacement. **A**–**J** showed the activation sequence of the “figure-of-eight” macro-reentry using a common isthmus, which was localized in a low voltage area, bounded by bilateral lines of block **K** at the postero-lateral wall of the RA. The white solid lines represented lines of block defined by the system algorithm. White arrows indicated the activation direction from the earliest to the latest area. In **L**, **M** the MAT was terminated by a short linear ablation across the common isthmus (LL and PA view). Note the small diastolic potentials at the site of MAT termination. In **N** a prophylactic short line was added to prevent other possible macro-reentries. Abbreviations referred to Fig. 1

Atriotomy related MAT was considered if the entire activation could be identified around the suture line/scar by high-density activation mapping and entrainment pacing from both sides of the suture line/scar produced a PPI matching the MAT-CL (Fig. 1D). “Figure-of-eight” reentry was considered if dual-loop reentry coexisted simultaneously with opposite propagation direction but shared a unidirectionally activated common isthmus [12] (Fig. 2).

### Mapping of critical isthmus for MAT

The critical isthmus for MAT was identified according to electroanatomic mapping as well as entrainment mapping [7, 10, 11], and was validated by MAT termination by subsequent RF ablation. On the voltage map, an area with bipolar voltage > 0.5 mV was defined as normal, ≤ 0.5 mV as low voltage area (LVA), and ≤ 0.05 mV as electrically silent area or scar (Fig. 1D). An area that could not be captured by high output pacing at 20 mA/2 ms pulse width or a line of wide double potentials separated by an isoelectric segment for more than 50 ms was also deemed as a scar [13]. The critical isthmus for MAT was usually located in low voltage areas, bounded by electrically silent areas or anatomic barriers, exhibiting fractionated, low amplitude, and long-duration mid-diastolic potentials [10–12] (Fig. 1D). Pacing entrainment at these sites could produce concealed fusion and a perfect PPI. If the CL was prolonged or the endocardial activation sequence had changed spontaneously or during ablation, conversion to another MAT was suspected and re-mapping was applied.

### Catheter ablation

A 3.5 mm saline-irrigated ablation catheter (Thermo-cool STSF, Biosense Webster) was applied for lesion creation. For CTI-dependent MAT, a linear lesion line was drawn between the tricuspid annulus and the inferior vena cava (IVC) at six o’clock direction from 45º left anterior oblique view (Fig. 1C). For non-CTI dependent MAT, continuous linear lesions were created at the critical isthmus of MAT (Fig. 1D). For peri-mitral MAT, endocardial linear ablation at lateral mitral isthmus (MI) was performed preferentially by connecting mitral annulus (MA) to left inferior pulmonary vein (PV). If endocardial ablation failed, epicardial ablation within the CS was applied (Additional file 1: Fig. S1C), or an anterior line was drawn by connecting MA to the anterosuperior ostium of right superior PV. For peri-mitral AFL, left-sided PV isolation could be performed ahead of MI ablation to form an electrical barrier. For MAT originating from PVs, the ipsilateral PVs were isolated. Bi-directional block of linear lesions was validated by differential pacing maneuvers (Additional file 1: Fig. S1D). For endocardial ablation, Radiofrequency (RF) power was delivered at 35–40 W, with a saline irrigation speed of 17 ml/min and an ablation index (AI) of 450–500. For epicardial ablation within CS, RF power 25 W, irrigation speed 30 ml/min, 30 s for each lesion was applied. The endpoint of ablation was abolishment and non-inducibility of all types of MAT by burst pacing at the shortest interval that could 1:1 capture the atrium from two separate sites (proximal CS and high right atrium).

### Follow-up

All patients were discharged after 2 days post-ablation. After discharge, patients were followed up at the out-patient clinic at 1, 3 months and then every 3 months. Transthoracic echocardiography (TTE) was performed at one-month post-ablation to rule out valve prosthesis malfunction. ECG and 24 h Holter monitoring were performed at 1, 3, 6, 12 months. Patients were asked to record an ECG if symptoms appeared indicating a recurrence. Therapeutic anticoagulation with warfarin was continued in patients with mechanical valve prosthesis, but could be discontinued 3 months post-ablation in ATa-free patients with bio-prosthetic valve and low risk of thromboembolism. No AADs were administered post-ablation, except in patients with recurrence or new development of AF. Clinical success was defined as freedom from recurrence of atrial tachy-arrhythmias (ATa) off AADs at the end of 12 months’ follow-up. Patients with MAT recurrence could undergo repeat ablation > 2 months after the index ablation.

### Statistical analysis

Continuous data with normal distribution were given as mean ± SD, or as median and range otherwise. Continuous data were compared by t test if the variance was equal, or by Mann–Whitney U test otherwise. Category data were given as counts or proportions (%), and were compared by Chi-square test or Fisher’s exact test when appropriate. The ATa-free survival probability in two groups was calculated by the Kaplan–Meier method and compared by Log-rank test. Potential predictors of ATa recurrence were evaluated by the univariate Cox proportional hazards modeling. A two-tailed *P* < 0.05 was considered statistically significant. Data processing and statistical analysis was performed by IBM SPSS Statistics 19.0 for Windows (IBM Corporation, Somers, NY, USA).

## Results

### Patients’ characteristics

There were 18 patients in TV group and 30 patients in MV group. In TV group annuloplasty ring was implanted in 12 and valve replacement was performed in the remaining 6 (1 with bio-prosthesis and 5 with mechanical prosthesis) due to secondary severe tricuspid insufficiency in 17 and Epstein’s anomaly in 1 patient. One patient with patent ductus arteriosus (PDA) underwent concomitant PDA ligation. In MV group, mitral valvuloplasty was performed in 1 and prosthetic valve replacement was performed in 29 (21 with mechanical prosthesis and 8 with bio-prosthesis) due to rheumatic or degenerative mitral valve disease. Four patients with coronary artery disease underwent concomitant coronary artery bypass grafting (CABG) surgery. One patient with ventricular septal defect (VSD) underwent concomitant VSD repair. The baseline demographic data was shown in Table 1. There was no significant difference in age, sex, AAD use, duration of MAT, time of MAT occurrence post-surgery, major comorbidities and echocardiographic measurements in two groups.

**Table 1:** The baseline demographic data in two groups

	TV group (n = 18)	MV group (n = 30)	P value
Male, n (%)	12 (66.7)	17 (56.7)	0.55
Age (years)	54.9 ± 14.1	56.8 ± 13.0	0.64
Duration of MAT (months)	3.0 (2.0, 8.25)	9.5(2.0, 26.3)	0.23
Time of MAT occurrence post-surgery	2.5 (0.0, 90.0)	12(0.0, 84.0)	0.83
AADs prior to ablation	1.7 ± 0.7	1.7 ± 0.8	0.87
Comorbidities			
Hypertension, n (%)	5 (27.8)	10 (33.3)	0.69
Diabetes Mellitus, n (%)	3(16.7)	5(16.7)	1.00
History of Stroke, n (%)	0	2 (5.7)	–
Concomitant cardiac defect, n (%)	Epstein’s anomaly 1 (5.5) PDA 1 (5.5)	CABG 4 (13.3), VSD 1(3.3)	0.60
TTE measurement			
LAD (mm)	48.3 ± 8.9	48.1 ± 5.6	0.91
LVEDD (mm)	46.8 ± 4.8	49.2 ± 6.4	0.17
LVESD mm)	32.4 ± 4.5	35.4 ± 7.1	0.12
Septum	10.0 ± 1.3	9.6 ± 2.7	0.60
RAD	44.8 ± 10.8	41.8 ± 4.4	0.18
RALD	51.7 ± 6.6	50.1 ± 3.7	0.30
LVPW	9.6 ± 1.7	9.0 ± 2.0	0.30
LVEF (%)	56.4 ± 8.5	52.0 ± 10.7	0.15

*MAT* macro-reentrant atrial tachycardia, *AAD* anti-arrhythmic drug, *LAD* left atrial diameter, *LVEDD* left ventricular end diastolic diameter, *LVESD* left ventricular end systolic diameter, *RAD* right atrial diameter, *RALD* right atrial longitude diameter, *LVPW* Left ventricular posterior wall, *LVEF* left ventricular ejection fraction

### Findings of electrophysiological mapping

The clinical arrhythmia was sustaining in all patients in TV group and in 29 patients in MV group, and was induced in the remaining 1 in MV group. The mean MAT-CL at presentation was comparable in two groups. Nineteen MATs were mapped in TV group, and the majority of which was CTI-independent AFL (84.2%). Of 16 CTI-dependent AFL, there were 11 counterclockwise and 5 clockwise macro-reentries. The other 3 non-CTI dependent MATs were located at RA free wall, at RA septum and LA roof, respectively. Thirty-nine MATs were identified in MV group. The proportion of CTI-dependent AFL (38.5%) declined dramatically in MV group than in TV group, *P* = 0.02. Of 15 CTI-dependent AFL, there were 8 counterclockwise and 7 clockwise macro-reentries. The other right atrial MATs were 8 suture line-related and 2 RA septum scar- related macro-reentries. Of 14 left atrial MATs, there were 8 peri-mitral flutter, 3 roof-dependent, 2 LA anterior scar-related, and 1 right PV (RPV)-related MAT.

There was no significant difference in mean number of MAT and the proportion of patients with ≥ 2 MATs in two groups. However, the proportion of patients with left atrial MAT was significantly higher in MV group than in TV group (*P* = 0.01), and the prevalence of left atrial MAT (14 / 39) was significantly higher in MV group than that (1 / 19) in TV group (*P* = 0.01). The summary of MAT mapping was shown in Table 2.

**Table 2:** The results of MAT mapping and ablation in two groups

	TV group (n = 18)	MV group (n = 30)	P value
MAT-CL at presentation (ms)	249 ± 28	250 ± 30	0.94
Number of MAT per patient	1.1 ± 0.3	1.3 ± 0.5	0.08
Proportion of Pts with ≥ 2 MATs, n (%)	2 (11.1)	9 (30.0)	0.13
Proportion of Pts with left atrial MAT, n (%)	1 (5.6)	12 (40.0)	0.01
Total number of MAT in each group	19	39	
Right atrial MAT, n (%)	18 (94.7)	25 (64.1)	
CTI-dependent AFL, n (%)	16 (84.2)	15 (38.5)	0.02
Counterclockwise	11	8	
Clockwise	5	7	
Non-CTI dependent MAT in RA			
RA lateral suture line-related MAT	1	8	
RA septum scar-related MAT	1	2	
Left atrial MAT, n (%)	1 (5.3)	14 (35.9)	0.01
Anterior LA scar related MAT	0	2	
Peri-mitral MAT	0	8	
LA roof dependent	1	3	
RPV related MAT	0	1	
Procedural duration (min)	85.5 ± 35.9	103.8 ± 44.6	0.14
Fluoroscopic time (min)	4.4 ± 1.3	6.3 ± 3.4	0.04
Acute success rate, n (%)	18 (100)	28 (93.3)	0.52

*MAT* macro-reentrant atrial tachycardia, *CL* clycle length

Concomitant atrioventricular nodal reentrant tachycardia was found in one patient in TV group following CTI-dependent atrial flutter ablation, which was successfully ablated by slow pathway modification.

### Results of CTI-dependent and peri-mitral flutter catheter ablation

The mean procedural duration, fluoroscopic time and acute success rate in two groups were shown in Table 2. The procedural duration and acute success rate were comparable between two groups, but the fluoroscopic time was shorter in TV group than that in MV group (*P* = 0.04).

First-pass CTI-dependent AFL termination and CTI block were achieved in 8 of 16 in TV group and in 13 of 15 in MV group, respectively, *P* = 0.03. In TV group, additional conduction gaps were ablated close to the prosthetic tricuspid annulus in 4, at the mid-portion pouch in 2. A more septal line connecting CS ostium with IVC was needed in 2. In MV group, additional conduction gaps were ablated at the mid-portion of CTI in 1 and at the ridge near the IVC ostium in 1. Bi-directional CTI block was validated in all patients by differential CS ostium pacing.

In MV group, endocardial ablation successfully blocked lateral MI in 3 of 8, and additional epicardial ablation within CS achieved MI block in 1. For the remaining 4 peri-mitral flutter, anterior line ablation terminated peri-mitral flutter in 3 (anterior line block failure in one) and fail to terminate AFL in 1.

### Ablation of MATs arising from other sites

In TV group, RA septal scar-related MAT was terminated by connecting the scar to the SVC (the critical isthmus) in 1, but it changed to LA roof-dependent MAT. The latter was terminated by LA roof linear ablation. RA lateral suture line-related MAT was terminated by connecting the suture scar to the IVC (the critical isthmus) in 1.

In MV group, of 8 RA lateral scar-related MAT, 6 were abolished by connecting the scar with the IVC (the critical isthmus), 1 by linear ablation extending from the scar to the SVC after failed ablation between the scar and the IVC (the critical isthmus defined by prior mapping), and 1 by connecting the scar with the crista terminalis (the critical isthmus). Two RA septum scar-related MATs were abolished by connecting the scar with the SVC (the critical isthmus). Two LA anterior scar-related MATs were abolished by connecting the scar with metal mitral annulus (the critical isthmus). Three LA roof-dependent MATs were abolished by roof line ablation. One RPV-related MAT was terminated by circumferential RPV isolation.

### Complications

Groin hematoma was found in 2 and 3 patients in TV and MV groups, respectively, and was treated conservatively. No other major complications occurred in both groups. There was no signs of prosthetic valve damage or malfunction by TTE check at one- month post-ablation.

### Follow-up

At the end of one year’s follow-up, 13 (72.2%) of 18 patients in TV group and in 23 (76.7%) of 30 patients in MV group were free of ATa recurrence, respectively, P = 0.70 (Fig. 1E). Univariate Cox proportional hazards regression analysis failed to find any significant factors that could predict ATa recurrence (Additional file 2: Table S1). In TV group persistent MAT in 4 and paroxysmal AF in 1 recurred after 1.7 ± 0.5 months post-ablation, four of whom underwent re-ablation. Recurrent CTI-dependent flutter in 2 was terminated by gaps ablation close to the prosthetic tricuspid annulus similar to the location of gaps during the index ablation. Peri-mitral flutter occurred in 1 case with previous CTI-dependent AFL ablation, but failed to be abolished. New onset of paroxysmal AF in 1 case was treated with the CPVI procedure. After repeat ablation, 16 of 18 patients were free of further ATa recurrence. In MV group, regular MAT in 6 (persistent in 5, paroxysmal in 1) and paroxysmal AF in 1 recurred after 2.1 ± 0.9 months post-ablation, six of whom underwent re-ablation. In 4 patients with recurrent peri-mitral AFL, gaps ablation in initial MI lines terminated the arrhythmia and exempted from further recurrence in 2, but ablation failed in the remaining 2. In 2 patients with previous RA lateral MAT ablation, new reentrant circuits in RA were terminated by linear ablation in 1 and no further recurrence was detected during follow-up. Paroxysmal MAT still recurred in the remaining 1. Altogether 26 patients were free of further recurrence following re-ablation.

## Discussion

### Major findings of this study

This report characterized the electrophysiological findings and one-year ablation outcomes of MAT in patients with tricuspid or mitral valve surgery. In contrast to TV group, MV group had significantly lower prevalence of CTI-dependent flutter, higher prevalence of left atrial MAT, higher proportion of patients with left atrial MAT. Nevertheless, the mid-term effectiveness of catheter ablation was comparable in both groups. No echocardiographic predictors for ATa recurrence were identified.

### Impact of cardiac valve surgery on the genesis of MAT

The pressure and volume overload in both atria could be alleviated by valve surgery, which might prompt atrial reverse remodeling and exert a beneficial effect on ATa occurrence. However, suture, atriotomy and scar formation might outweigh the favorable effect of valve surgery and provide a substrate for MAT [2]. Moreover, different surgical LA access approaches had significant impact on the mechanisms of post-surgical MAT. Dual-loop MAT might occur following transseptal incision for mitral valve repair [8]. Left atriotomy might result in scar formation at the postero-septal wall close to the right-sided PVs, and prompt genesis of macro-reentry utilizing LA roof as its critical isthmus [7].

In this study, we found the predominant type of MAT was CTI-dependent AFL and ablation in the RA might be enough in the vast majority of patients in TV group. Other non-CTI dependent right atrial MATs were infrequent and usually located at RA lateral wall or septum, where suture lines or scar provided the substrate for macro-reentries. Left atrial ablation was hardly required except in a patient utilizing a LVA at LA roof as the substrate for reentry. The ablation results in TV group in our study were consistent with the findings of a previous study [4].

In contrast, In MV group, the prevalence of left atrial MATs significantly increased, and peri-mitral flutter was the most common type of left atrial MATs. The other left atrial MATs could be found at LA anterior wall, roof and PV, all of which were scar-related macro-reentries. These results were in accordance with the findings of the previous studies [6–8]. Due to the complex substrate in MV group, frequent conversion from one type of MAT to another is likely to occur during ablation, and comprehensive bi-atrial mapping might be necessary before the mechanisms of MATs can be clarified.

### Technical considerations for CTI and MI linear ablation

CTI linear ablation, by connecting the tricuspid anulus with the IVC, was a standard practice for the treatment of typical AFL, with a high acute and long-term success rate [14, 15]. However, in the setting of tricuspid valve surgery, CTI ablation might be extremely challenging due to altered anatomy, tissue folding and potential risks of prosthetic valve damage and catheter entrapment. The scar contracture might lead to displacement of tricuspid annulus and deformities of the CTI area. Surgical sewing of annuloplasty ring or valve prosthesis might cause tissue folding, which rendered some critical areas inaccessible or unable to be penetrated by RF energy [3]. In our study, we found the first-pass rate of typical AFL termination and CTI block in TV group was significantly lower than that in MV group. Of note, the conduction gaps in TV group were mostly close to prosthetic tricuspid annulus. Whereas in MV group, they were mostly close to the mid-portion of CTI and IVC ostium. Although CTI was initially blocked by gaps ablation at the prosthetic tricuspid valve in 2 patients, typical AFL recurred and necessitated repeated ablation at the same area. In a previous study, ablation at the ventricular side of the bio-prosthetic valve was needed to interrupt typical AFL after TV replacement. However, this approach was contraindicated in those with mechanical valve prosthesis [3].

The aforementioned obstacles also existed in peri-mitral AFL ablation after mitral valve surgery. In our series, although peri-mitral AFL was terminated in 7 of 8 in the index procedure, it recurred in 4 during subsequent follow-up, and re-ablation only succeeded in 2 of them. Additional epicardial ablation within the CS was useful to block MI, but this approach might be limited by the CS anatomy and carried potential risks of coronary artery injury. Anterior line ablation was an alternative approach; however, it also involved ablation close to the mitral valve prosthesis, hence RF energy penetration might be compromised due to tissue folding at mitral annulus, and the risks of prosthetic valve damage and catheter entrapment could not be excluded.

### Ablation of MATs other than CTI-dependent and peri-mitral AFL

Suture line or scar-related MATs might be encountered during the procedure in both groups. The reentrant circuits could be identified precisely by electroanantomic and entrainment mapping maneuvers. Ablation at the critical isthmus between two anatomic barriers or electrically silent areas was usually effective to interrupt these MATs. Despite the high acute success rate, late recurrence of MAT or even new onset of AF might not be excluded due to formation of new reentrant circuits and the progression of atrial disease. In our series, new right atrial MATs occurred in 2, and new AF onset was observed in 2. In such cases, an approach comprising empirical multiple linear ablations to block all possible reentrant pathways might be plausible. In cases of AF onset, circumferential PV isolation and other adjunctive approaches should be applied as appropriate.

### Predictors of MAT recurrence

In our study we could not find any echocardiographic predictors for MAT recurrence. Left and right atrial enlargement seemed not to predict MAT recurrence after ablation. This result was in accordance with the finding of a previous report [4]. In that study, the dimension and function of RA and right ventricle were not independent predictors of ATa recurrence after AFL ablation in patients with Epstein’s anomaly. This finding might be interpreted by the fact that the predominant type of AT after valve surgery was macro-reentries around tricuspid annulus, mitral annulus and surgical scar/suture lines. In most patients, linear ablation at CTI, MI or other critical isthmus might be enough to prevent further recurrence if the linear lesions were reliably and permanently blocked [4, 11], irrespective of the atrial or ventricular size.

## Limitations

This study should be acknowledged to have several limitations. Due to the retrospective design, data collection, frequency and time interval of patient visit might vary individually. Due to the limited valve surgery volume in our institute, the number of patients enrolled in TV group was relatively small, so extrapolation of the findings of our study to generalized population with post-valve surgery MAT should be made with caution. The findings in TV group should be testified in large-scale, prospective and multi-center trials. Moreover, Implantable event recorder or trans-telephonic ECG monitoring was not applied in this study, thus short or asymptomatic episodes could have been missed and the ablation success rate might have been overestimated. Because the follow-up period was set to be 12 months, the long-term performance of catheter ablation for treating post-surgical MAT could not be clarified.

## Conclusions

In conclusion, the types of MAT varied significantly in patients with prior tricuspid or mitral valve surgery; However, the mid-term effectiveness of catheter ablation for MAT was comparable in two groups.

## Supplementary Information

**Additional file 1: Figure S1. **Peri-mitral flutter ablation after mechanical mitral valve prosthesis replacement. In **A**, **B** entrainment pacing at LA anterior wall and lateral mitral isthmus (MI) produced a perfected PPI (6 ms and 0 ms exceeding the cycle length of the MAT [260 ms], respectively) with paced CS activation sequence identical to that of MAT, which was consistent with peri-mitral flutter. In **C** MAT was terminated by combined endocardial ablation and epicardial ablation within the CS. In **D** MI conduction block was validated by pacing at left atrial appendage. The CS activation sequence was from proximal to distal and the interval from the pacing artifact to CS 1,2 was 350 ms, indicating that MI was blocked (PentaRay positioned at high right atrium). LL left lateral view, *MAT* macro-reentrant atrial tachycardia, *CL* cycle length, *PPI* post-pacing interval, *LA* the left atrium, *CS* coronary sinus.

**Additional file 2: Table S1. **Univariate Cox regression analysis for ATa recurrence.

## Data Availability

The datasets used and/or analyzed during the current study are available from the corresponding author on reasonable request.

## Abbreviations

MAT: Macro-reentrant atrial tachycardia
AT: Atrial tachycardia
AF: Atrial fibrillation
TV: Tricuspid valve
MV: Mitral valve
PPI: Post-pacing interval
CTI: Cavo-tricuspid isthmus
CS: Coronary sinus
MI: Mitral isthmus
TTE: Transthoracic echocardiography
AAD: Antiarrhythmic drugs
ATa: Atrial tachy-arrhythmia
