# Supplementary figures and images for: Macro-reentrant atrial tachycardia after tricuspid or mitral valve surgery: is there difference in electrophysiological characteristics and effectiveness of catheter ablation?
Source: BMC Cardiovasc Disord. 2021 Nov 12;21:538. doi: 10.1186/s12872-021-02368-w (PMC8588703; doi:10.1186/s12872-021-02368-w)

## Slide 1
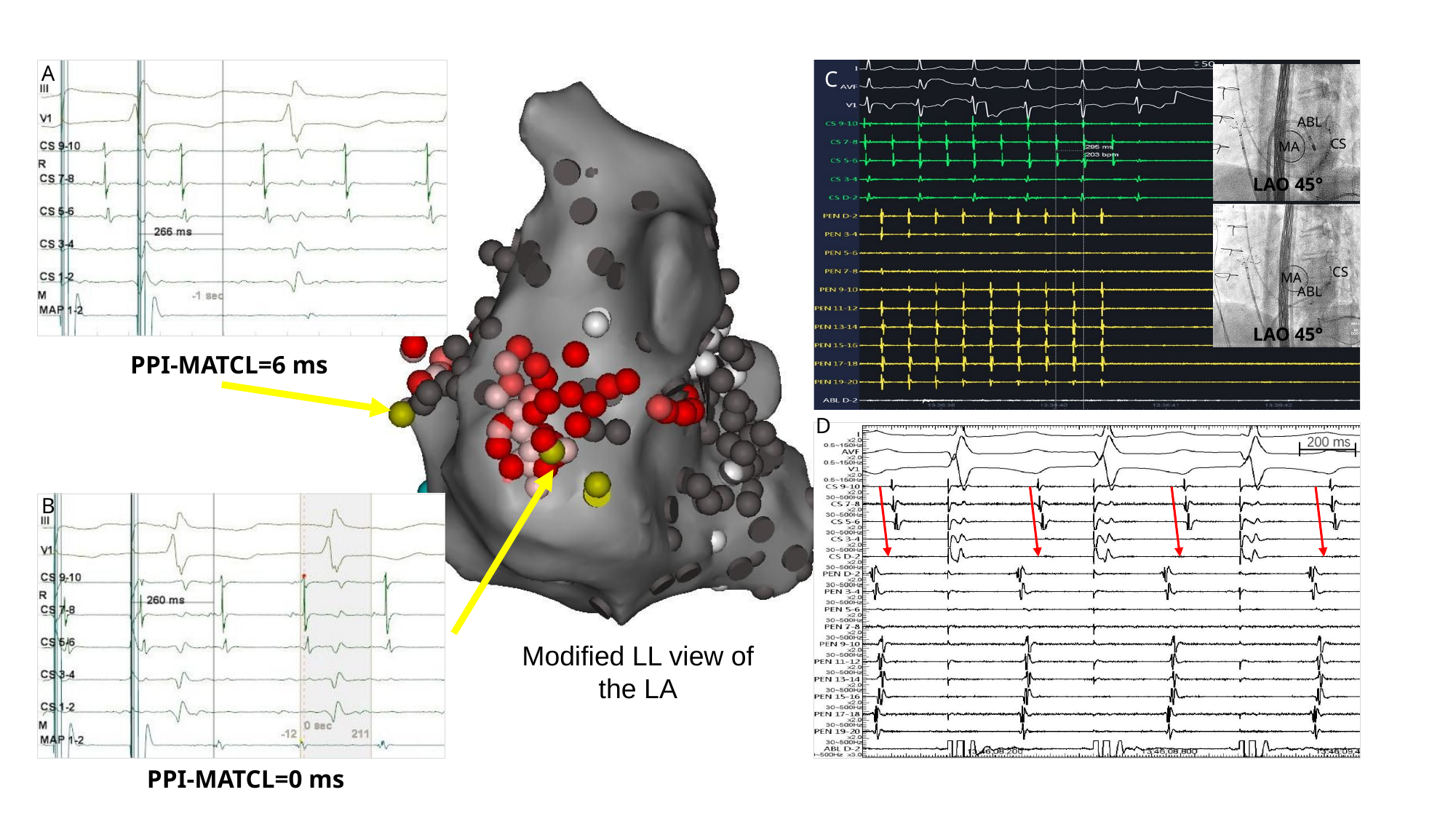

A
C
ABL
CS
MA
LAO 45°
CS
MA
ABL
LAO 45°
PPI-MATCL=6 ms
D
B
Modified LL view of the LA
PPI-MATCL=0 ms

Supplement: Supplementary file 1 — Additional file 1: Figure S1. Peri-mitral flutter ablation after mechanical mitral valve prosthesis replacement. In A, B entrainment pacing at LA anterior wall and lateral mitral isthmus (MI) produced a perfected PPI (6 ms and 0 ms exceeding the cycle length of the MAT [260 ms], respectively) with paced CS activation sequence identical to that of MAT, which was consistent with peri-mitral flutter. In C MAT was terminated by combined endocardial ablation and epicardial ablation within the CS. In D MI conduction block was validated by pacing at left atrial appendage. The CS activation sequence was from proximal to distal and the interval from the pacing artifact to CS 1,2 was 350 ms, indicating that MI was blocked (PentaRay positioned at high right atrium). LL left lateral view, MAT macro-reentrant atrial tachycardia, CL cycle length, PPI post-pacing interval, LA the left atrium, CS coronary sinus. [file 12872_2021_2368_MOESM1_ESM.pptx]
