# Supplementary material for: Macro-reentrant atrial tachycardia after tricuspid or mitral valve surgery: is there difference in electrophysiological characteristics and effectiveness of catheter ablation?
Source: BMC Cardiovasc Disord. 2021 Nov 12;21:538. doi: 10.1186/s12872-021-02368-w (PMC8588703; doi:10.1186/s12872-021-02368-w)
Supplement: Supplementary file 2 — Additional file 2: Table S1. Univariate Cox regression analysis for ATa recurrence. [file 12872_2021_2368_MOESM2_ESM.pptx]

## Slide 1
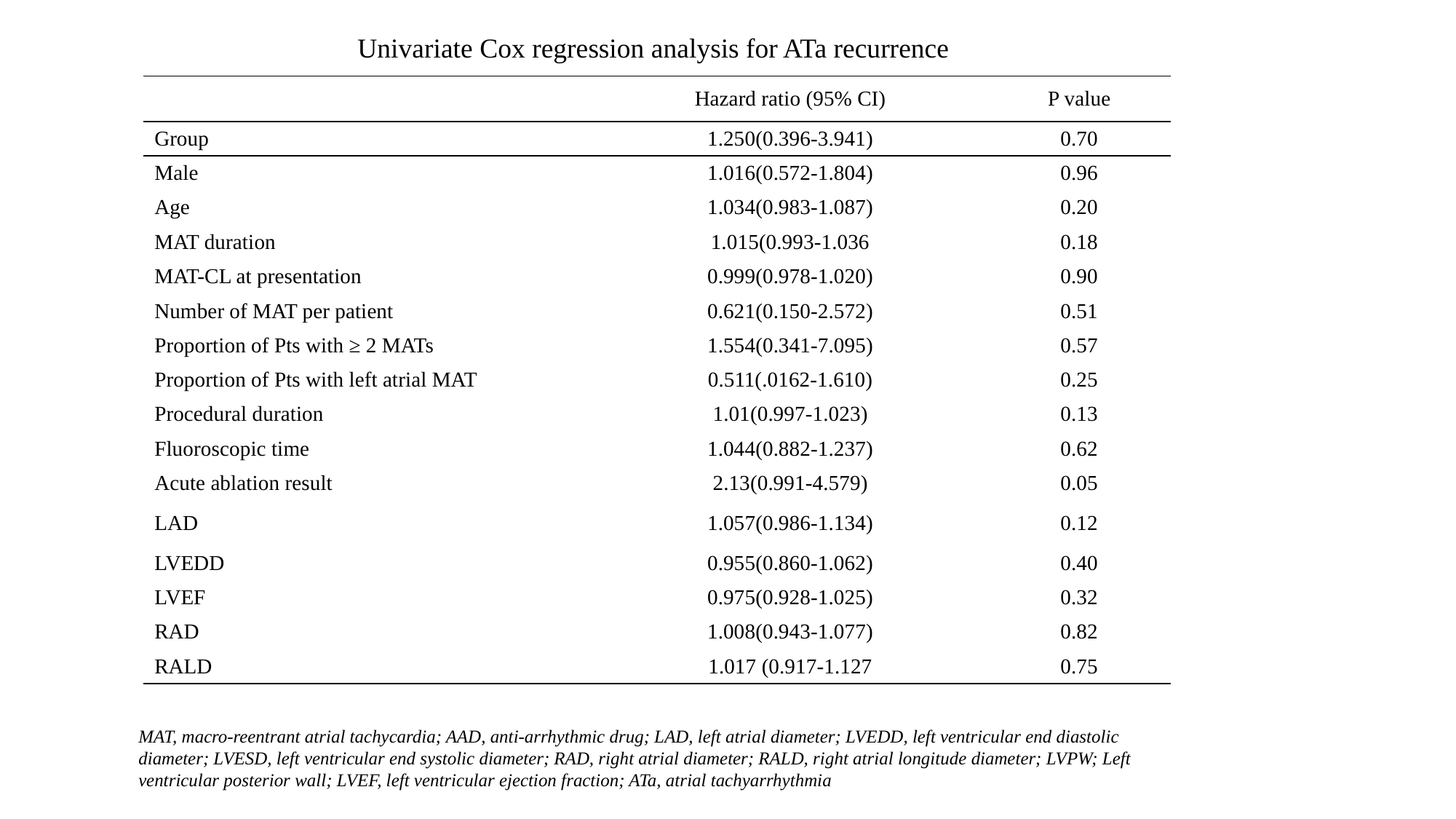

Univariate Cox regression analysis for ATa recurrence
| | Hazard ratio (95% CI) | P value |
| --- | --- | --- |
| Group | 1.250(0.396-3.941) | 0.70 |
| Male | 1.016(0.572-1.804) | 0.96 |
| Age | 1.034(0.983-1.087) | 0.20 |
| MAT duration | 1.015(0.993-1.036 | 0.18 |
| MAT-CL at presentation | 0.999(0.978-1.020) | 0.90 |
| Number of MAT per patient | 0.621(0.150-2.572) | 0.51 |
| Proportion of Pts with ≥ 2 MATs | 1.554(0.341-7.095) | 0.57 |
| Proportion of Pts with left atrial MAT | 0.511(.0162-1.610) | 0.25 |
| Procedural duration | 1.01(0.997-1.023) | 0.13 |
| Fluoroscopic time | 1.044(0.882-1.237) | 0.62 |
| Acute ablation result | 2.13(0.991-4.579) | 0.05 |
| LAD | 1.057(0.986-1.134) | 0.12 |
| LVEDD | 0.955(0.860-1.062) | 0.40 |
| LVEF | 0.975(0.928-1.025) | 0.32 |
| RAD | 1.008(0.943-1.077) | 0.82 |
| RALD | 1.017 (0.917-1.127 | 0.75 |
MAT, macro-reentrant atrial tachycardia; AAD, anti-arrhythmic drug; LAD, left atrial diameter; LVEDD, left ventricular end diastolic diameter; LVESD, left ventricular end systolic diameter; RAD, right atrial diameter; RALD, right atrial longitude diameter; LVPW; Left ventricular posterior wall; LVEF, left ventricular ejection fraction; ATa, atrial tachyarrhythmia
